# Comparison of the accuracy of different slot properties of 3D-printed cutting guides for raising free fibular flaps using saw or piezoelectric instruments: an in vitro study

**DOI:** 10.1007/s11548-025-03474-2

**Published:** 2025-07-12

**Authors:** Britta Maria Lohn, Stefan Raith, Mark Ooms, Philipp Winnand, Frank Hölzle, Ali Modabber

**Affiliations:** Department of Oral and Maxillofacial Surgery, University Hospital RWTH Aachen, Pauwelsstraße 30, 52074 Aachen, Germany

**Keywords:** Computer-aided design, Surgical instruments, Fibula, Mandible/surgery, Reconstructive surgical procedures, Surgical planning, Reproducibility of results

## Abstract

**Purpose:**

The free fibular flap (FFF) is a standard procedure for the oral rehabilitation of segmental bone defects in the mandible caused by diseases such as malignant processes, osteonecrosis, or trauma. Digital guides and computer-assisted surgery (CAS) can improve precision and reduce the time and cost of surgery. This study evaluates how different designs of slot cutting guides, guiding heights, and cutting instruments affect surgical accuracy during mandibular reconstruction.

**Methods:**

Ninety model operations in a three-part fibular transplant for mandibular reconstruction were conducted according to digital planning with three guide designs (standard, flange, and anatomical slots), three guide heights (1 mm, 2 mm, 3 mm), and two osteotomy instruments (piezoelectric instrument and saw). The cut segments were digitized using computed tomography and digitally evaluated to assess surgical accuracy.

**Results:**

For vestibular and lingual segment length, the anatomical slot and the flange appear to be the most accurate, with the flange slightly under-contoured vestibularly and the standard slot over-contoured lingually and vestibularly (*p* < 0.001). There were only minor differences between the use of saw and piezoelectric instrument for lingual (*p* = 0.005) and vestibular (*p* < 0.001) length and proximal angle (*p* = 0.014). The U-distance after global reconstruction for flanges resulted in a median deviation of 0.0468 mm (IQR 8.15), but was not significant (*p* = 0.067).

**Conclusion:**

Anatomical slots and flanges are recommended for osteotomy, with guiding effects relying on both haptic and visual control. Unilateral guided flanges also work accurately at high guidance heights. The results of piezoelectric instrument (PI) and saw showed comparable results in the assessment of individual segments and U-reconstruction in this in vitro study without soft tissue, so that the final decision is left to the expertise of the surgeons.

**Supplementary Information:**

The online version contains supplementary material available at 10.1007/s11548-025-03474-2.

## Introduction

Major bony defects in the mandible can lead to segmental bone defects, posing significant challenges for surgical reconstruction. The osteomyocutaneous free fibula flap is the workhorse for bony reconstruction. It was first performed for mandible reconstruction by Hidalgo in 1989 with alternatives such as scapula or pelvic bone harvesting [1, 2].

Conventional reconstruction of the mandible with a free fibular flap (FFF) involving manual measuring and prebending of a lead template is a complex procedure requiring considerable surgical experience. Preoperative three-dimensional (3D) imaging with computed tomography (CT) scans are used for digital planning and the production of models and sawing templates [3–5].

According to Jewer et al. [6], in the case of a mandibular LCL defect (L = lateral, C = central), i.e., an angle-to-angle defect of the mandible, a 3-segment reconstruction is recommended, with a minimum size of 20 mm required to maintain adequate blood flow [6–8].

The digital planning and fabrication of cutting guides has significantly enhanced the precision of FFF raising. Segmental resection of the lower jaw can impair chewing, swallowing, breathing, and speaking. The optimal curvature of the lower jaw can be reconstructed using an algorithm proposed by Raith et al. [9], which is based on the individual bone curvatures of the mandible and fibula and automatically generates valid reconstruction proposals.

Digital planning with computer-assisted surgery (CAS) offers opportunities to reduce ischemia and improve graft survival [10, 11]. This protects patients from unnecessarily long anesthesia and reduces costs associated with surgery. Osteotomy templates accelerate and simplify complex surgeries.

The accuracy of the transfer of planned mandibular reconstructions depends on two steps. First, precise osteotomy of the fibula segments must consider both length and angle. Second, it is crucial to ensure the exact alignment of the segments for U-shaped reconstruction. Cutting guides are frequently used to ensure the accurate transfer from intensive planning to surgery [11–13]. Various templates have been described in technical papers and tested for accuracy [14–18], yet it remains unclear which design yields the best results.

Dong et al. [19] examined the accuracy of cuts made in synthetic bone with different slot widths, without considering the effect of different instruments. Their approach involved manual repositioning for gluing without screws. However, this could result in uncontrolled manipulation when connecting segments.

We are not aware of any study in which the manual application of the segments is completely omitted; thus, the true error or surgical accuracy of different guide variants can be measured. This study evaluates how different designs of slot cutting guides, guiding heights, and cutting instruments affect surgical accuracy during mandibular reconstruction.

## Materials and methods

### Virtual surgical planning

The original CT dataset of the Synbone fibula (*Fibula medium left 1565*) was used alongside a patient’s skull CT scan and converted into Digital Imaging and Communications in Medicine (DICOM) format. To achieve realistic segmentation of the plastic bones, each fibula analog underwent zinc coating prior to the CT scan. The data were uploaded to AC-Seg for segmentation with the help of artificial intelligence (AI) [20]. A standardized defect dimension was defined by simulating an LCL defect according to Jewer et al. [6].

A three-segment fibula reconstruction was planned with the specialized in-house developed software suite AC-Plan, that is implemented in Python programming language and taking advantage of functionalities of the 3D computer graphics software Blender (Version 3.5, Blender Foundation, Amsterdam, The Netherlands).

**Fig. 1:**
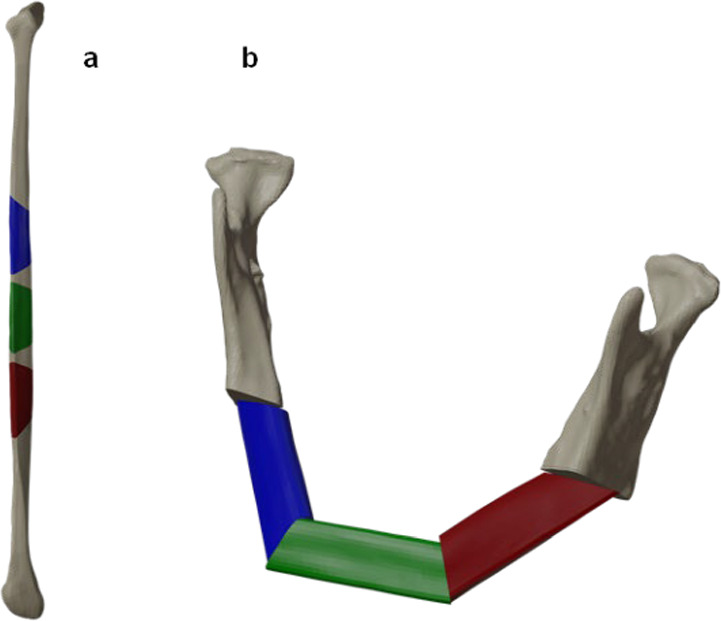
** a** Left fibula with three marked segments **b**, reconstructed mandible with three fibula segments

Medical engineers designed three guide forms for three-segment fibular reconstruction (Fig. 1): standard slots (SS), flanges (FS), and anatomical slots (AS). In addition to guides, various slot heights were tested: 1 mm, 2 mm, and 3 mm.

**Fig. 2:**
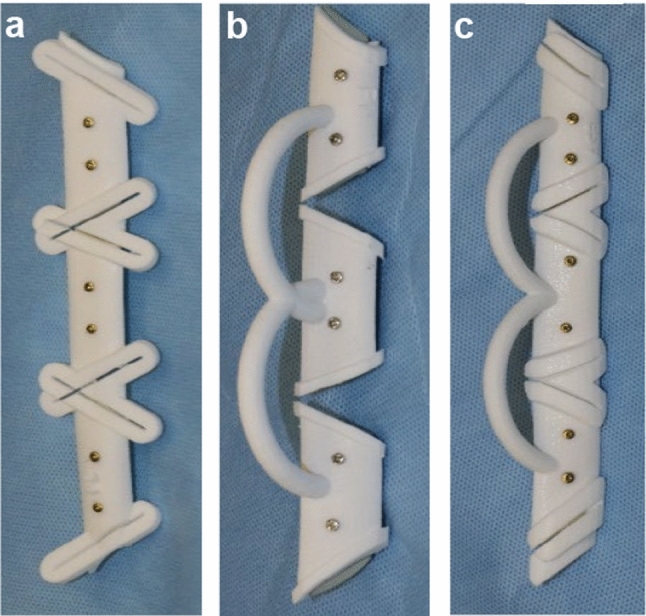
Laser-sintered cutting guides for free fibular flap for three-segment mandibular reconstruction **a** standard slot (SS), **b** flange (FS), **c** anatomical slots (AS)

A guide opening of 1 mm was selected for a working width, 0.3 mm for the oscillating saw, and 0.45 mm for the piezoelectric instrument (PI). During the model operation on the plastic fibula allograft without soft tissue, we omitted the standard offset of 2 mm between the guide and the fibula bone. Thus, the guides had a material thickness of 2 mm to prevent bending during surgery. The guides were exported as STL-files and printed with PROTIQ (Blomberg, Germany) using a powder-bed-based 3D laser sintering process from PA-12 material (Fig. 2).

Each guide was planned at a distance of 7 cm from the lateral malleolus based on clinical assessment. Accordingly, the guide was fixed with two fixation screws per segment at a distance of 9 cm from the most caudal point of the fibula to prevent tilting and rotation during the procedure.

### Model operations

All model surgeries on 90 fibula analogs were performed by an experienced surgeon trained in pilot studies using instructions and artificial bone models. Each guide—AS, SS and FS—was used to perform the osteotomy on 30 randomized fibulae. The procedures were repeated five times per design combination for surgical instruments and guide heights to account for learning curves or fatigue.

### Data evaluation

**Fig. 3:**
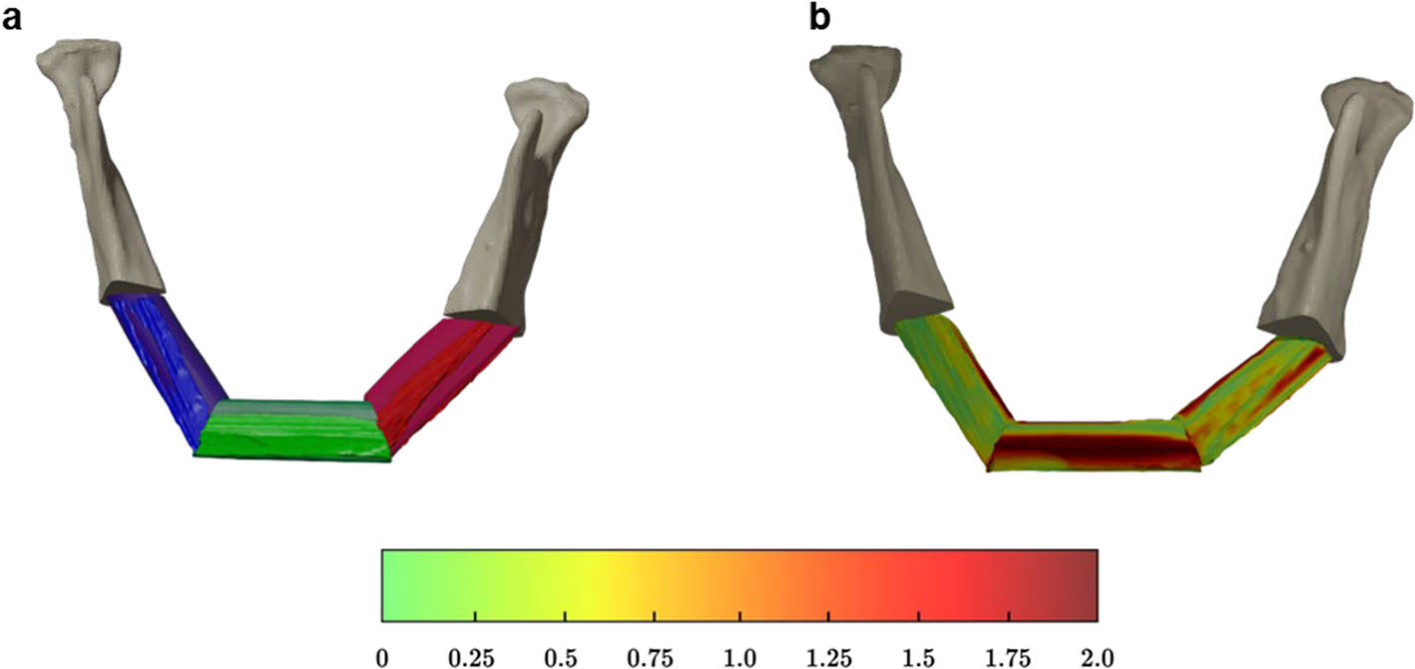
** a** Best-fit alignment of the planned and executed osteotomies and U-reconstruction. **b** Heatmap showing deviation of the digital mandibular reconstruction with the VSP according to the color coding (in mm)

The model surgery was performed with a saw and PI on 90 artificial fibula bones. Grafts were digitized using CT scans and segmented using the thresholding tool from AC-Seg. Using the 3D software Blender (version 3.5, Blender Foundation, Amsterdam, The Netherlands), the osteotomy surfaces were marked on the segmented surfaces and the osteotomy planes digitally created and geometrically evaluated as described by Winnand et al. [21]. Using the planes, digital mandibular reconstruction was conducted with the best-fit alignment between scans and digital planning. The heat map visualizes the color-coded discrepancies from planning (in mm) (Fig. 3a, b).

**Fig. 4:**
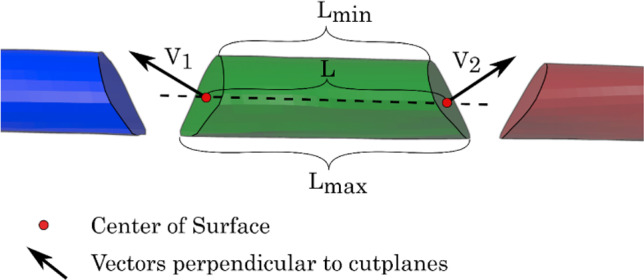
Visualization of the minimal segment length (*L*_min_, lingual side for reconstruction) the maximal segment length (*L*_max_, vestibular side for reconstruction) and the normal vectors on the cutting surfaces (*V*_1_ and *V*_2_)

Each segment’s maximum and minimum length (Fig. 4), underwent evaluation alongside proximal/distal angles based on leg positioning references. Fusion of the segments resulting in the neomandible were compared via U-distance, reflecting the distance between the two most laterally exposed points.

### Statistical analysis

Differences between virtual surgical planning (VSP) and results from the model operation concerning distances and angles were determined using the Friedmann test or Wilcoxon signed rank test. Independent groups were compared using the Kruskal–Wallis test. The significance level was *p* < 0.05. All analyses were performed with SPSS Statistics (version 27; IBM Corp.) and GraphPad Prism (version 10; GraphPad Software Inc.).

## Results

All surgical guides were utilized in combination with the oscillating saw and the PI to create three segments in each specimen without complete failures, culminating in a total of 540 conducted model osteotomies.

### Evaluation using basic criteria: slot design

**Fig. 5:**
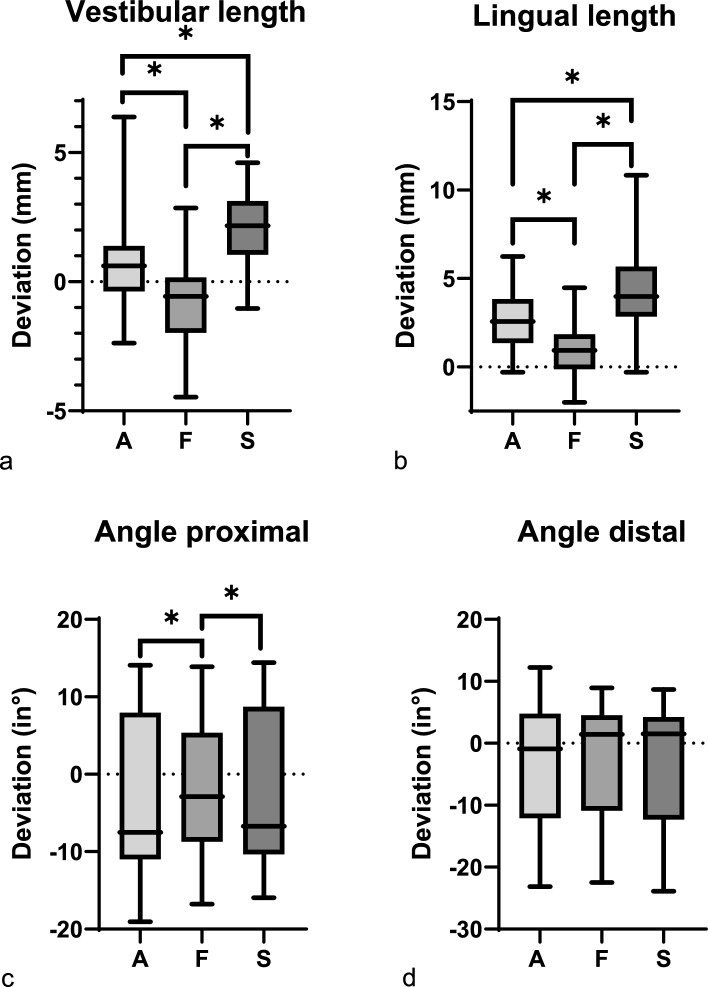
The deviation in distances (in mm) and angles (°) from the digital planning is presented in Tukey’s box-and-whisker plots (*p* < 0.05 for a, b, c; *p* > 0.05 for d). *p* values corresponding to testing for differences between reality and VSP in terms of distances and angles using Friedman rank test for paired samples. *p* values corresponding to testing for differences between different types of slots ((standard slot (SS), flange (FS) and anatomical slot (AS)) are marked (* = *p* < 0.05). The line in the middle of each box represents the median for **a** vestibular length, **b** lingual length, **c** angle proximal and **d** angle distal of all segments

The deviations from digital planning for different slot types are shown in Fig. 5. Significant differences from digital planning were observed for lingual length (*p* < 0.001), vestibular length (*p* < 0.001), and proximal angle (*p* = 0.008).

### Evaluation using basic criteria: instrument

**Fig. 6:**
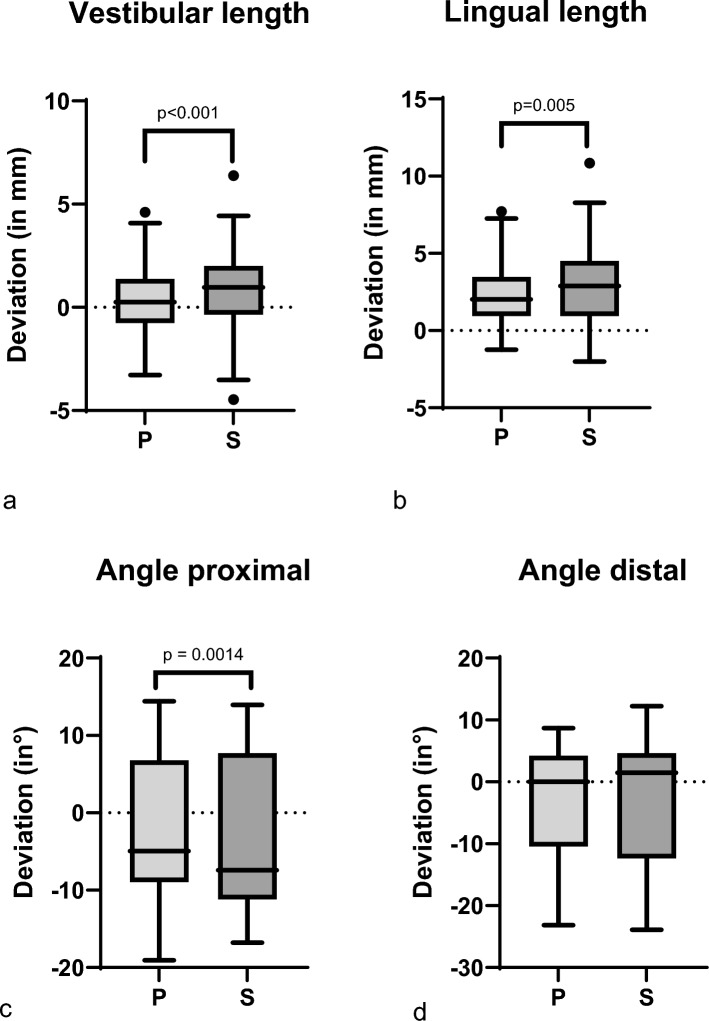
The deviation in distances (in mm) and angles (°) from the digital planning is presented in Tukey’s box-and-whisker plots (*p* < 0.05 for a, b, c; *p* > 0.05 for d). *p* values corresponding to testing for differences between reality and VSP in terms of distances and angles using paired samples Wilcoxon rank test for osteotomy instruments (P = Piezo instrument; S = Saw). The line in the middle of each box represents the median for **a** vestibular length, **b** lingual length, **c** angle proximal and **d** angle distal of all segments

Analysis revealed significant differences across all segments between saw and PI for lingual length (*p* = 0.05), vestibular length (*p* < 0.001), and proximal angle (*p* = 0.014), albeit with negligibly small amounts (Fig. 6).

### Evaluation using basic criteria: guiding height

**Fig. 7:**
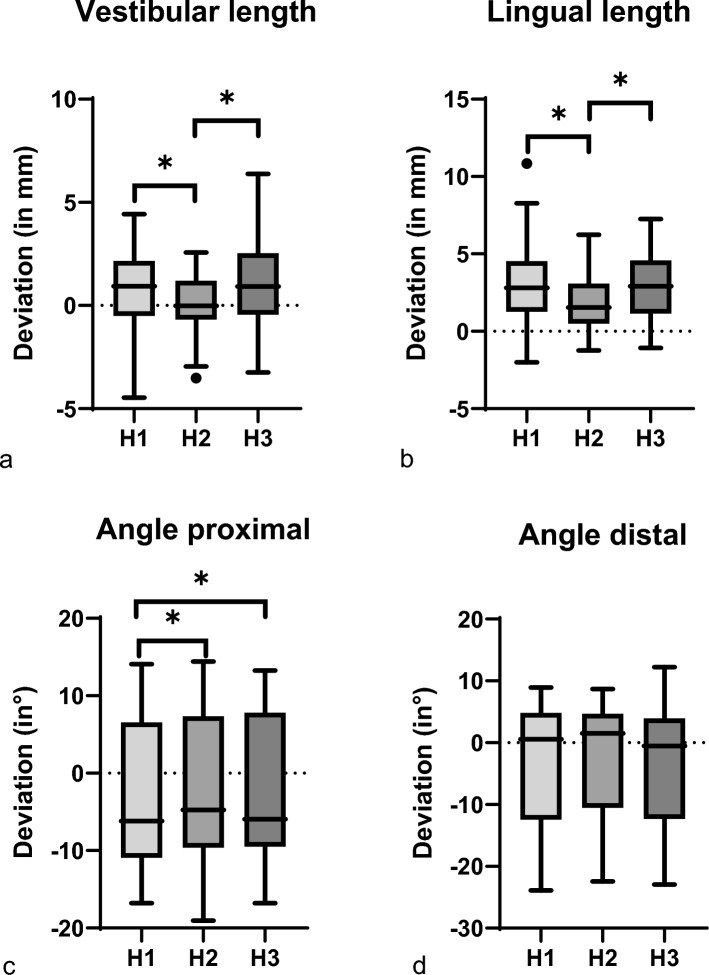
The deviation in distances (in mm) and angles (°) from the digital planning is presented in Tukey’s box-and-whisker plots (*p* < 0.05 for a, b, c; *p* > 0.05 for d). *p* values corresponding to testing for differences between reality and VSP in terms of distances and angles using Friedman rank test for paired samples. *p* values corresponding to testing for differences between different slot heights (H1 = 1mm; H2 = 2mm; H3 = 3mm) are marked (* = *p* < 0.05). The line in the middle of each box represents the median for **a** vestibular length, **b** lingual length, **c** angle proximal and **d** angle distal of all segments

The guiding height also displayed patterns with significant differences noted for linear deviation (*p* < 0.001) and proximal angle (*p* = 0.011), disregarding the slot type and the osteotomy instrument (Fig. 7).

### Evaluation of the single segment accuracy

**Fig. 8:**
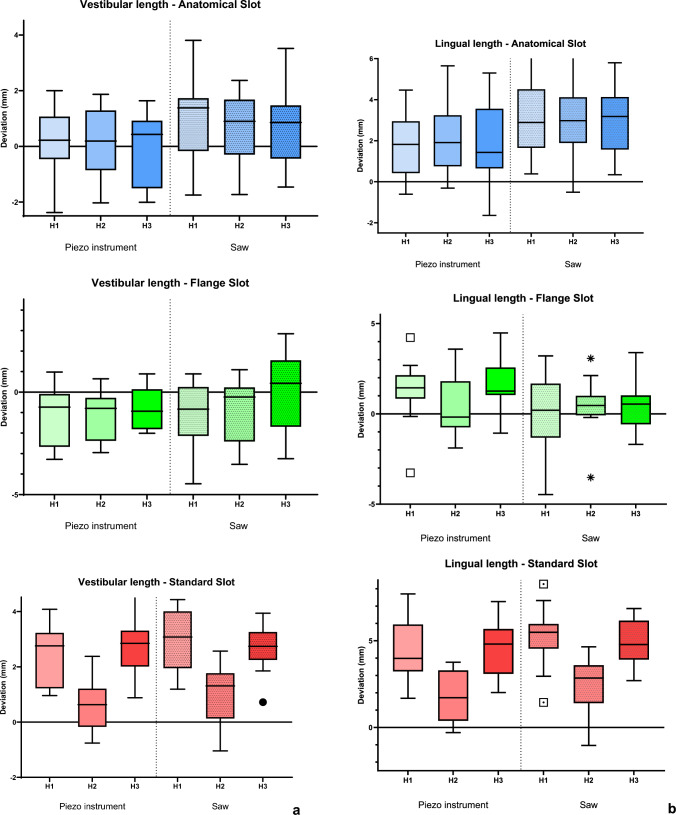

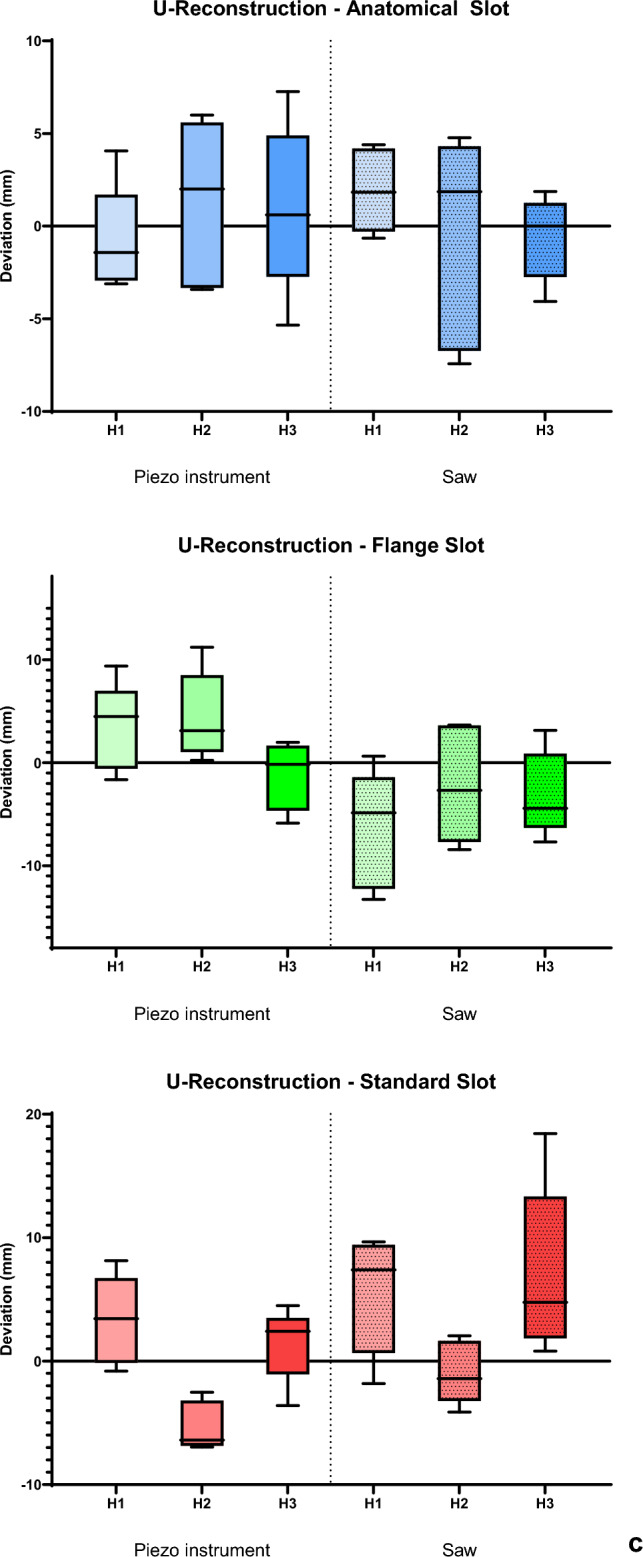
The deviation (in mm) from the digital planning, considering the combined effect of slot design (AS, FS, SS), varying height (1, 2, or 3 mm), and the choice of instrument (piezo or saw), is presented in Tukey’s box-and-whisker plots (*p* < 0.05 for a, b; *p* > 0.05 for c), separated for each guide design. The line in the middle of each box represents the median for **a** vestibular length, **b** lingual length of each segment, and **c** U-distance of the mandibular reconstruction (*n* = 5)

The comparison of independent groups per fibula segment (*n* = 270) indicated significant differences (*p* < 0.05) related to linear deviation at vestibular and lingual length (Fig. 8a, b). Angular deviations were not statistically significant (*p* > 0.05). The data showing the deviation from planned segment length (vestibular and lingual) and segment angle (proximal and distal) for the individual segments are attached in the supplemental information (SI 2).

### Devaluation of the global reconstruction

After the fusion of all segments, the AS exhibited slight deviations in the U-distance regardless of guide height or instrument type used for osteotomy (U-distance [in mm] Median (IQR): AS 0.3463 (6.73); FS 0.0468 (8.15); SS 1.7545 (7.28); *p* = 0.067), as shown in Fig. 8c.

## Discussion

FFF remains the most favored transplant for mandibular reconstruction in the case of large bone defects. The difficulty lies in the redesign of a triangular tubular bone into a round shape in the ideal position of the mandibular contour [22]. Features of a successful reconstruction include the osteosynthesis with bone contact, good intersegmental bone-to-bone contact, and ensuring the minimum size of 20 mm for safe healing [7, 23]. The plan must be implemented accurately so that the mandibular contour is achieved, and a precise transition to the fixation on the ascending mandibular ramus is possible without pathological deflection of the ascending ramus [24, 25]. Building on the importance of accurate reconstruction, digital planning is a critical tool for achieving optimal functional and aesthetic outcomes in mandibular reconstructions. The use of cutting guides ensures high accuracy during surgery for later oral rehabilitation, reduced ischemia time, and improved graft survival [10, 26], thereby reducing intraoperative planning time [27].

The fundamental challenge in maxillofacial reconstruction is the precise transfer of digital planning to the surgical site. Consistent with this objective, Dong et al. [19] examined different slot width effects on fibular reconstruction accuracy using fibula analogs. The authors could not find a significant difference in segment length. The groove restriction effect described by Dong et al. [19] indicated the maximum deflection in the vertical direction leading to increased angular deviations of the osteotomy planes depending on the slot width. However, the groove restriction effect also influences the vestibular segment length, the fibula guide connecting surface, in the horizontal dimension. With a slot width of 1 mm, we considered both restrictions for the AS and SS. Considering the results for vestibular length, there were significant differences, and it appeared that the restriction caused by the guide had the expected effect on the AS. However, due to the lack of bilateral guidance, it did not work with the undersized segments using FS.

This study investigated the effect of groove restriction in the vertical dimension using different guide heights, which were intended to enable haptic depth control by pressing the instrument against the guide height [28, 29]. To illustrate, supplemental information (SI 1) is attached. It was hypothesized that the maximum guide height (3 mm) would allow the least deviation. Notably, a guide height of 2 mm yielded an accurate result for vestibular segment length—suggesting that visual guidance may be limited at greater heights (3mm) while insufficient haptic feedback occurs at lower heights (1mm). The FS with 3 mm guidance is an exception. It can be assumed that a greater height does not lead to a visual restriction due to the one-sided guidance, and therefore, the segment length is more accurate with higher guidance.

To date, no standardized instruments exist for performing osteotomies. The more expensive PI reduces the risk of accidental damage to the vascular pedicle, which is not considered in the artificial fibula used here. In this study, all osteotomy variants were performed with a PI and an oscillating saw, with the PI being superior, albeit by a small amount. The slight superiority of the PI could be explained by its slower execution and thus better visual control and corrective action in this in vitro model. The execution time was not examined in this study. However, in pilot runs, the oscillating saw appeared to be clearly superior to the PI. Furthermore, in real surgical situations, osteotomy instruments, especially PI, are often reinserted to complete the osteotomy, which can lead to additional inaccuracies in the osteotomy plane. On the other hand, the saw also benefits from the in vitro situation without soft tissue. The piezo instrument is advantageous for osteotomies close to the pedicle. For clinical translation, no clear recommendation can be made for the operating theater; the choice of instrument is left to the surgeon.

The existing literature shows that, when all segments are considered, the accuracy of digital planning for FS transmission exceeds the standards of other studies [21, 30, 31], which reported a total linear deviation of 1.3–1.9 mm. It is noteworthy that other authors [5, 32, 33], reported an accuracy of less than or equal to 1.0 mm. However, a small number of cases lacked a standardized mandibular defect or fibula segment number.

As a significant contribution to the existing literature, this study shifts the focus from single segments to the assessment of the alignment accuracy for the U-shaped mandibular reconstruction, consciously avoiding manual positioning or fixation with adhesive. We emphasize the precise measurement of distal horizontal distance, which is crucial for an effective transfer from planning to alignment of the residual jaw without interfering with the remnant jaws. For reconstruction using patient-specific implants (PSI), the accurate production of the vestibular bone surface, which initially serves as support for the guide, is crucial for a precise fitting of the customized plate [34]. If the osteotomy plane angles are aligned correctly, anterior overlap at the bone junction can occur due to oversized single vestibular segments but can be later reduced for contouring. Over-dimensioning of the lingual length is particularly problematic, as it has a greater blocking effect on the U-reconstruction and consequently on the position of the remaining ascending jaw.

While there is no significant difference in the distal angle of the fibula segments for different slot types, the difference in proximal angle is more pronounced. FS with the least vestibular and lingual deviation also exhibits significantly lower angular deviations at the proximal angle. In contrast, the AS, which shows slight overcontouring vestibularly but is notably oversized lingually by + 2.5 mm, demonstrates a significant deviation at the proximal angle. This discrepancy may impact the overall U-reconstruction, where FS show minimal deviation, while the AS exhibits greater deviation.

However, precise osteotomy alone is not sufficient for accurate transfer of the digital planning. Precise positioning of the guide is also a prerequisite for an accurate osteotomy results [35]. Depending on the positioning, the fibula shape differs. Varying fibula thickness results in different lingual segment lengths due to the angled osteotomy prescribed by the guiding slot. This affects the precise U-reconstruction of the neomandible.

Certain limitations apply to this in vitro study. Our model does not include soft tissue on the fibula analogs. Based on in vitro tests, the effect of visual guidance is superior to haptic guidance. However, since the FFF has a vascular pedicle and is favorably raised with a skin island, visibility of the guiding groove and the osteotomy itself may be limited. Therefore, haptic guidance might be more advantageous than visual guidance in a real operating theater. Although the involvement of only one experienced surgeon limits the generalizability of the results, we exclude the influence of individual osteotomy techniques, preferences, and surgical experience on the osteotomy results in order to achieve standardization for consistent results. Besides deviations from the planning, which result from inaccuracies/variables in the implementation, there is also a generalized inaccuracy in the use of the CT dataset, segmentation, and 3D-printing [36].

Knowledge gained from this in vitro study might be transferred to the clinic. Transferring the accuracy of the distal horizontal U-distance as the point of contact with the residual mandible might be interpreted as interfering contacts and provoking undesirable deflection of the remnant jaw compared to virtual planning. Future studies could include a patient-specific implant with pre-drilled holes indicating the position of the osteosynthesis plate on the remaining ramus, as they are already used on the patient [34]. However, large-scale production remains costly and is not feasible for in vitro studies like this involving many samples. A finite element model (FEM) could be considered as an alternative for further investigation of osteotomy differences through different slot variations or even for simulation of the load on different performed osteosynthesis between the segments and the remaining jaw, as it does not require real patients or high case numbers, but only a data set, e.g., a CT data set, which is analyzed mathematically [37–40].

An additional disadvantage is that planning and surgical implementation are inflexible. Intraoperative correction of the mandibular resection borders is not possible. Studies have already been conducted on the transfer accuracy of digital planning with surgical navigation [41, 42] or augmented reality (AR) [21, 26, 43]. The tracking method for verifying the guide position appears promising [44, 45], but no equivalent results have yet been achieved for osteotomy using AR. In the next step, future studies including soft tissue and clinical conditions are needed. Osteosynthesis between both the segments and the remaining jaws could address the limitations of this study and more realistically assess the effects on the accuracy of mandibular reconstructions [39, 40]. AR guidance may enhance this process, potentially employing a collaborative laser system [46, 47], to provide real-time feedback in the region of the segmental osteotomy of the lower jaw for tumor cells, with consecutive adjustments of the osteotomy on both the lower jaw and fibula.

Based on this study’s results, using the FS to achieve optimal accuracy is recommended. Although digital planning with CAD-CAM-fabricated guides for oral rehabilitation in mandibular segment resections is both planning- and cost-intensive, it offers greater precision in reconstructions while reducing surgical risks, ultimately benefiting patients who are already significantly burdened.

## Conclusion

Based on the study results, the following recommendations for the use of AS and FS are offered. If depth control does not improve with high guides, the guiding effect is a combination of haptic and visual control. Since the results of PI and saw showed no great difference in the consideration of the individual segments or in the U-reconstruction, the choice is left to the surgeons’ experience.

## Supplementary Information

Below is the link to the electronic supplementary material.

Supplementary file1 (PDF 176 kb)

## Abbreviations

3D: Three dimensional
AI: Artificial intelligence
AR: Augmented reality
CAS: Computer-assisted surgery
CT: Computed tomography
DICOM: Digital Imaging and Communications in Medicine
FEM: Finite element model
FFF: Free fibular flap
LCL: L = lateral, C = central, Angle-to-angle mandibular defect, Jewer’s classification
PI: Piezoelectric instrument
PSI: Patient-specific implant
VSP: Virtual surgical planning
