# Supplementary material for: Comparison of the accuracy of different slot properties of 3D-printed cutting guides for raising free fibular flaps using saw or piezoelectric instruments: an in vitro study
Source: Int J Comput Assist Radiol Surg. 2025 Jul 12;20(12):2501–12. doi: 10.1007/s11548-025-03474-2 (PMC12689674; doi:10.1007/s11548-025-03474-2)
Supplement: Supplementary file 1 — Supplementary file1 (PDF 176 kb) [file 11548_2025_3474_MOESM1_ESM.pdf]

International Journal of Computer Assisted Radiology and Surgery

COMPARISON OF THE ACCURACY OF DIFFERENT SLOT PROPERTIES OF 3D-PRINTED CUTTING GUIDES FOR RAISING FREE FIBULAR FLAPS USING SAW OR PIEZOELECTRIC INSTRUMENTS: AN IN VITRO STUDY

Britta M. **Lohn**[*;1]; Stefan **Raith**[1]; Mark **Ooms**[1]; Philipp Winnand[1]; Frank **Hölzle**[1]; Ali **Modabber**[1]

1 Department of Oral and Maxillofacial Surgery, University Hospital RWTH Aachen, Pauwelsstraße 30, D-52074, Aachen, Germany

***Corresponding author:**

Britta Maria Lohn
Department of Oral and Maxillofacial Surgery, University Hospital RWTH Aachen
Pauwelsstraße 30
D-52074 Aachen, Germany
Phone: 0049 - 241 – 8088258
Fax: 0049-241-8082430
E-Mail: blohn@ukaachen.de

SUPPLEMENTARY INFORMATION

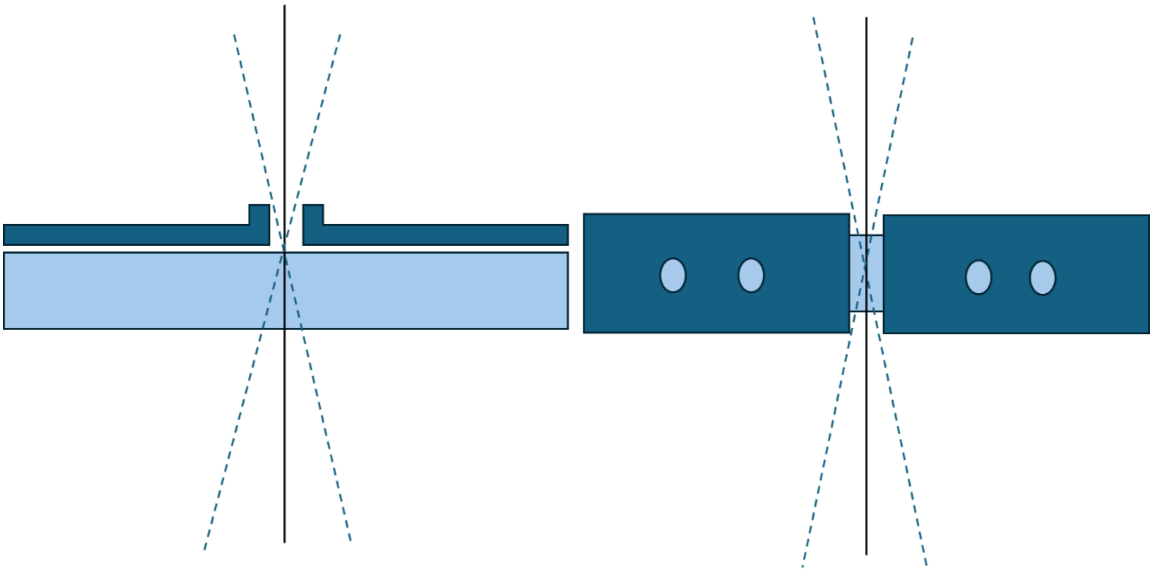

SI 1. The groove restriction effect shows the maximum deflection (blue dashed lines) along the slot opening of the guide (dark blue) compared to the optimal osteotomy (black solid line) of the fibula segments (light blue) **a** Angular deviation of the osteotomy due to deflection in the vertical dimension, described by Dong et al. [21] **b** linear deviation in the horizontal dimension, which led to size deviation on the vestibular side towards the guide

SI 2. Deviations in segment length and segment angle for different guides

Mode	Lingual length (in mm) Median (IQR)	Vestibular length (in mm) Median (IQR)	Angle distal (in °) Median (IQR)	Angle proximal (in °) Median (IQR)
ASP1	1.8217 (2.37)	0.2159 (1.53)	0.0355 (17.7)	-3.1755 (18.01)
ASP2	1.9084 (2.44)	0.1939 (2.15)	-0.1602 (14.8)	-3.8191 (15.08)
ASP3	1.9089 (2.67)	0.4252 (2.43)	-1.9465 (19.36)	-5.5208 (18.79)
ASS1	2.8924 (3.56)	1.3780 (1.90)	-0.7703 (20.79)	-10.1797 (20.32)
ASS2	2.9881 (1.91)	0.9021 (1.99)	1.3405 (15.25)	-9.1837 (23.49)
ASS3	3.1797 (2.23)	0.9304 (1.98)	-1.1418 (2.25)	-8.6102 (19.57)
FSP1	1.4415 (1.04)	-0.7329 (2.57)	-0.6747 (20.34)	-4.2221 (13.35)
FSP2	-0.1848 (2.56)	-0.7879 (2.10)	0.6213 (13.08)	-2.3475 (9.79)
FSP3	1.8635 (1.51)	-0.9261 (1.95)	-3.8307 (17.14)	-5.5602 (14.67)
FSS1	0.2045 (3.0)	-0.8299 (2.39)	3.1799 (20.35)	-3.2485 (10.31)
FSS2	0.4998 (0.70)	-0.2442 (2.64)	3.2465 (18.12)	-1.9390 (14.79)
FSS3	0.6848 (0.86)	0.4273 (3.24)	3.9109 (15.67)	-0.4707 (15.58)
SSP1	4.1849 (2.46)	2.7569 (2.01)	1.5515 (15.40)	-7.7901 (19.72)
SSP2	1.7090 (2.90)	0.6324 (1.38)	1.2829 (14.69)	-6.7558 (22.43)
SSP3	4.8099 (2.43)	2.8534 (1.30)	-2.4904 (18.53)	-6.3722 (17.33)
SSS1	5.6690 (1.69)	3.0830 (2.06)	0.5396 (20.42)	-10.9069 (20.05)
SSS2	2.8526 (2.19)	1.3106 (1.64)	-2.5051 (20.39)	-10.7326 (21.26)
SSS3	5.5684 (2.22)	2.7385 (1.01)	0.6938 (17.57)	-9.1132 (20.54)

Data presented as median (with interquartile range) for distances (mm) and angles (°) testing for differences between different guide designs respecting all combinations of slot variation (standard slot (SS), flange (FS) and anatomical slot (AS)), height variation (1mm, 2mm, 3mm)

and instrument (Piezo (P) and saw (S)) in terms using Kruskal-Wallis test ($p<0.05$ for linear deviations, $p>0.05$ for angular deviations)
